# Diabetes with kidney injury may change the abundance and cargo of urinary extracellular vesicles

**DOI:** 10.3389/fendo.2023.1085133

**Published:** 2023-04-03

**Authors:** Dongfeng Gu, Yanan Ding, Xin Jiang, Beili Shen, Luca Musante, Harry Holthofer, Hequn Zou

**Affiliations:** ^1^ Department of Nephrology and Transplantation Center, Zhengzhou People’s Hospital, Henan University of Chinese Medicine, Zhengzhou, China; ^2^ Division of Nephrology and Center for Immunity, Inflammation and Regenerative Medicine, Department of Medicine, University of Virginia, Charlottesville, VA, United States; ^3^ Department of Medicine, University Medical Center Hamburg-Eppendorf, Hamburg, Germany; ^4^ Department of Nephrology, South China Hospital of Shenzhen University, Shenzhen, China

**Keywords:** urinary extracellular vesicles, diabetes, diabetic nephropathy, diabetes with microalbuminuria, diabetes with macroproteinuria

## Abstract

**Background:**

Urinary extracellular vesicles (uEVs) are derived from epithelia facing the renal tubule lumen in the kidney and urogenital tract; they may carry protein biomarkers of renal dysfunction and structural injury. However, there are scarce studies focusing on uEVs in diabetes with kidney injury.

**Materials and methods:**

A community-based epidemiological survey was performed, and the participants were randomly selected for our study. uEVs were enriched by dehydrated dialysis method, quantified by Coomassie Bradford protein assay, and adjusted by urinary creatinine (UCr). Then, they identified by transmission electron microscopy (TEM), nanoparticle track analysis (NTA), and western blot of tumor susceptibility gene 101.

**Results:**

Decent uEVs with a homogeneous distribution were finally obtained, presenting a membrane-encapsulated structure like cup-shaped or roundish under TEM, having active Brownian motion, and presenting the main peak between 55 and 110 nm under NTA. The Bradford protein assay showed that the protein concentrations of uEVs were 0.02 ± 0.02, 0.04 ± 0.05, 0.05 ± 0.04, 0.07 ± 0.08, and 0.11 ± 0.15 μg/mg UCr, respectively, in normal controls and in prediabetes, diabetes with normal proteinuria, diabetes with microalbuminuria, and diabetes with macroproteinuria groups after adjusting the protein concentration with UCr by calculating the vesicles-to-creatinine ratio.

**Conclusion:**

The protein concentration of uEVs in diabetes with kidney injury increased significantly than the normal controls before and after adjusting the UCr. Therefore, diabetes with kidney injury may change the abundance and cargo of uEVs, which may be involved in the physiological and pathological changes of diabetes.

## Introduction

Over the past three decades, diabetes and prediabetes are significantly increased nationwide among children, adolescents, and younger adults (1). The estimated overall prevalence of prediabetes and diabetes were 10.9% and 35.7%, respectively, in Chinese adults (2). Most importantly, half of diabetic patients do not know that they have diabetes. Adults and children with impaired fasting glucose and/or impaired glucose tolerance were considered as prediabetes cases since they have not met the criteria of a diabetes diagnosis. Before presenting with obvious clinical symptoms, prediabetes may have complications, even if these were not observed. According to the survey of 1999–2016 in America, youth with prediabetic levels of HbA1c or fasting glucose were a high-risk population prone to developing cardiometabolic diseases (3). Hyperglycemia and diabetes are rising globally, and they are the most common cause of chronic kidney disease, and diabetic nephropathy is the major cause of end-stage renal disease (4). Therefore, more and more attention should be drawn to diabetes and its complications.

Extracellular vesicles (EVs) are secreted by cells of all tissues and organs under normal and disease conditions, ranging in size from approximately 30 to 10,000 nm in diameter and containing surface receptors, membrane and soluble proteins, lipids, ribonucleic acids, and genomic and mitochondrial DNAs (5–7). Urinary extracellular vesicles (uEVs) are derived from epithelia throughout the urogenital tract, podocyte, and transitional epithelia from the urinary collection system and released into the urinary lumen. Glomerular, tubular, prostate, and bladder cells are the most common sources of these vesicles (8). The uEV excretion is related to estimated glomerular filtration rate, creatinine clearance, total kidney volume, and kidney weight; one document revealed that nephrectomy may reduce uEV excretion, but depending on the loss of nephron mass (9). Therefore, 99.96% of the proteins presenting in uEVs may be the characteristic of cells under normal and disease conditions as assessed by proteomic analysis (8), and uEVs are being explored for non-invasive biomarkers (10, 11) of kidney function, kidney disease, and urological disease by proteomic and transcriptomic analyses, such as acute kidney injury (12), chronic kidney disease (13), polycystic kidney disease (14), prostate cancer (15), and renal allograft rejection (16, 17).

uEVs may be involved in the progress of diabetic nephropathy because they carry valuable sources for disease-stage-specific information and have the natural quality of fingerprints in disease progression (18). However, there are scarce data reported about the application of uEV research in different stages of diabetes (19). Therefore, we perform this study in order to research the physiological and pathological secretion of uEVs in healthy controls and diabetes patients with and without kidney injury.

## Materials and methods

### Participants

**Figure 1:**
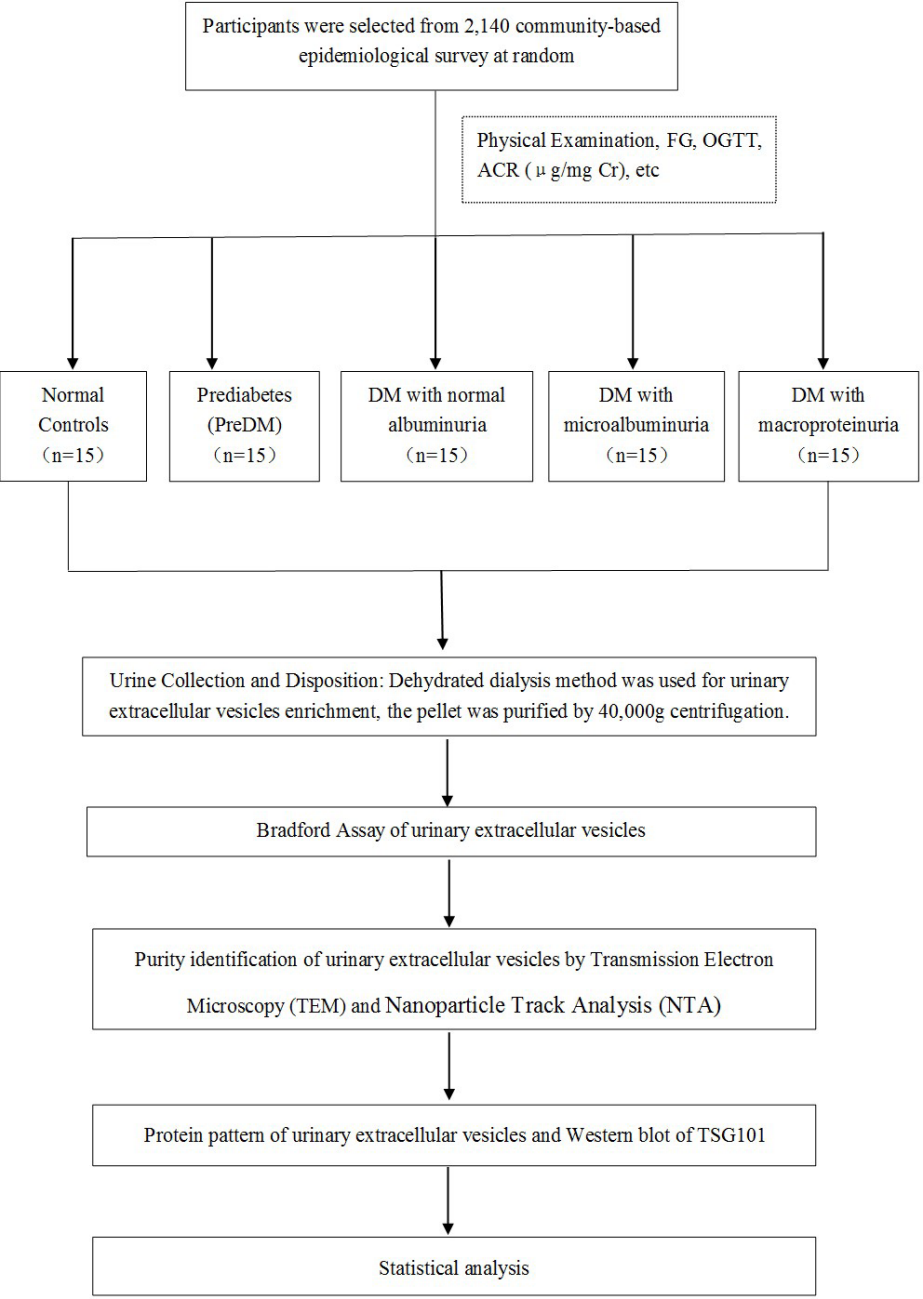
A schematic representation of the methodology.

A schematic representation of the methodology is shown in **Figure 1**. **A** total of 75 participants coming from a community-based screening in Zhuhai, Southern China, were randomly selected in our study (20, 21). The selected participants were separated into five groups: healthy controls (*n* = 15), prediabetes (*n* = 15), diabetes with normal proteinuria (*n* = 15), diabetes with microalbuminuria (*n* = 15), and diabetes with macroproteinuria (*n* = 15). Written informed consent prior to data collection was given to all participants. Leaflets were read to the illiterate participants, followed by a thumb impression. This study was approved by the Human Ethics Committee of the Third Affiliated Hospital of Southern Medical University, Guangzhou, China (20, 21).

### Demographic characteristics, anthropometric measurements, and urine and blood collection

All participants successfully filled in the questionnaire, which included data on age, sex, personal and family history of disease, waist circumference, height, and three readings of blood pressure taken at 1-min intervals. The first morning midstream urine without proteases inhibitor was collected from all the participants. Women who had their menstrual period must be excluded, but they are included once their menstruation is over. Blood was drawn on an empty stomach at local community clinics or health stations. The blood samples were disposed as soon as possible or, if there was a delay, stored at 4°C for less than 2 days (22, 23). Immunoturbidimetric test was carried for albuminuria examination. Jaffe’s kinetic method was used for UCr examination. Examinations on urinary albumin-to-creatinine ratio (ACR; μg/mg creatinine), fasting serum insulin concentration, serum creatinine, serum total cholesterol, high-density lipoprotein cholesterol, triglycerides (TG), and low-density lipoprotein cholesterol were performed simultaneously. Because the urine samples must be disposed in the central laboratory, they were transported with dry ice and then stored at -80°C (24).

### Evaluation criteria

Normal controls were selected from healthy participants, who should be non-smokers and non-drinkers and had no history of hypertension, dyslipidemia, central obesity, cardiovascular disease, stroke and tumor, infectious disease, and other kinds of disease. Prediabetes was defined as fasting plasma glucose (FPG) in the range 5.6–6.9 mmol/L (100–125 mg/dl) or OGTT 2 h after eating in the range 7.8–11.1 mmol/L (140–200 mg/dl). An empty stomach was defined as 8 h without eating. FPG ≥7.0 mmol/L (≥126 mg/dl) or OGTT 2 h after eating greater than 11.1 mmol/L (200 mg/dl) was defined as diabetes (25). ACR lower than 30 μg/mg in the spot urine of diabetes was defined as diabetes with normal proteinuria, ACR in the range of 30–299 μg/mg in the spot urine of diabetes was defined as diabetes with microalbuminuria, and ACR greater than 300 μg/mg in the spot urine of diabetes was defined as diabetes with macroproteinuria (25, 26).

### uEV enrichment

A hydrostatic filtration dialysis method was used for uEV enrichment, which was established based on our previous method (27). First, fresh urine or thawed urine from -80°C was centrifuged at 2,000 *g* of relative centrifugal force for 30 min, and then the supernatant was collected. Second, a 1,000-kDa nanomembrane (Spectrum Laboratories, Inc., CA, USA) was used for dehydrated dialysis in order to discard the soluble proteins in the supernatant at 2,000 *g*. The dehydrated dialysis was stopped when the remaining urine volume in the tube was approximately 3–5 ml, and then the urine in the tube was collected. Dialyzed urine was experienced at 40,000 *g* centrifugation (Beckman JA-25.15; Beckman Coulter, CA, USA) for 1 h, and the pellet at 40,000 *g* was collected and finally suspended in ultra-pure water.

### Bradford assay of uEVs

Coomassie Protein Assay Kit (Thermo Scientific, IL, USA) was used for the Bradford assay (28) according to the manufacturer’s instructions. The content of one ampule of bovine serum albumin (BSA) was diluted into six clean vials with ultra-pure water by twofold serial dilutions; thus, we got the standard concentrations of 25, 50, 100, 200, 400, and 800 μg/ml. Coomassie Reagent Solution was mixed immediately by gently inverting the bottle a couple of times. An equilibrated amount of reagent was removed at room temperature before use. Next, a 20-μl standard, unknown samples, and ultra-pure water as blank were pipetted into a 96-well plate; this was repeated three times each. Each well was added with 200 μl Coomassie Reagent and then mixed with a plate shaker for 30 s. Incubation for 10 min at room temperature carried out after this progression. The absorbance was detected at 595 nm by SpectraMax M5 Multi-Mode Microplate Readers (Molecular Devices LLC, CA, USA).

### Identification of uEVs by TEM

uEVs were identified by TEM. The Formvar-coated electron microscopy grid was held with forceps and washed gently with a drop of (10 μl) 0.01% BSA. After a while, a filter paper was used to suck from the edge of the grid. Then, a 2-μl sample was drawn immediately onto the grid and left on for 5 min, and a filter paper was used to suck from the edge of the grid. The grid was stained with 10 μl of 3% phosphotungstic acid (w/v) for 1 min, and then a filter paper was used again to suck from the edge of the grid. The grid was put directly into the grid box and then air-dried for half an hour before observation. After that, uEVs were identified by TEM (Hitachi H-7650; Hitachi, Tokyo, Japan).

### Identification of uEVs by NTA

NanoSight NS300 equipped with sCMOS camera was used for uEV analysis (NanoSight Ltd., Salisbury, UK). NTA Version 2.3 Build 0033 was used throughout. All experiments were carried out at 1:200 dilution factor, except for diabetes with macroproteinuria group with 1:1,600, and 0.25 ml was loaded for each sample. An aliquot of 20 μl of each sample of the same group was mixed together as a one-go experiment, and five group experiments were performed.

### Western blot of TSG101

Western blot of tumor susceptibility gene 101 (TSG101) was conducted according to our previous report (29–31). Specifically, 5-μg protein samples were loaded on resolving gels. After sodium dodecyl sulfate polyacrylamide gel electrophoresis, the proteins were transferred to a polyvinylidene difluoride membrane and then saturated with 5% BSA–phosphate-buffered saline (PBS) solution. The membrane was incubated with rabbit anti-human TSG101 antibody (Sigma Aldrich, Dorset, UK), followed by 6 times of washing with PBS-0.1% Tween, and then incubated with the appropriate horseradish peroxidase-conjugated secondary antibody (Dako, Ely, UK). After another round of six times of washing, the membrane was incubated with a detection reagent for 30 s and then visualized by Kodak IS 4000R image station (Kodak, USA).

### Statistical analysis

A one-way ANOVA with *post hoc* analysis was used for multiple comparisons of the basic characteristics and protein concentration of uEVs among five groups by SPSS 16.0. A *P*-value less than 0.05 was considered as significant difference. Graphs were created by GraphPad Prism 5.0.

## Results

### Baseline information of normal controls and diabetes patients

The characteristics of normal controls and diabetes patients are shown in **Table 1**. One-way ANOVO revealed that there was a significant difference in between group variation of blood glucose, albumin-to-creatinine ratio, systolic blood pressure, heart rate, serum creatinine, and triglycerides. There was no significant difference in between group variation of sex, age, diastolic blood pressure, urine creatinine, blood urea nitrogen, waist circumference, and total cholesterol.

**Table 1:** Characteristics of participants in different groups.

	NC	Pre-DM	DM	DM- micro	DM-macro	P value
Sex -male%	37.5%	43.7%	37.5%	50%	45.5%	P>0.05
Age -Year	40.75±11.1	61.75±1.31	57.62±7.89	60.81±12.05	65.63±12.59	P>0.05
Blood Glucose -mmol/l	4.64±0.36	6.00±0.43	6.23±1.49	8.59±3.27	6.78±2.18	P<0.001
ACR -μg/mg	6.70±2.71	10.67±5.64	11.39±6.42	71.26±55.64	380.57±61.74	P<0.001
SBP -mmHg	114.53±12.93	131.72±13.19	132.00±14.89	140.77±20.43	154.61±25.82	P<0.001
DBP-mmHg	74.22±10.33	77.09±9.62	82.03±9.06	77.73±6.53	79.55±12.44	P>0.05
HR - beats/min	74.62±7.99	78.40±6029	79.84±6.59	74.25±9.92	88.62±20.50	P<0.001
SCr –μmol/l	68.43±11.67	78.68±15.53	70.56±13.18	80.33±15.69	87.09±35.31	P<0.05
UCr –μmol/l	12.74±6.99	11.19±5.40	9.01±3.65	9.08±4.34	10.22±4.68	P>0.05
BUN –mmol/l	4.48±0.88	5.04±0.98	5.52±1.32	6.09±2.02	6.42±2.38	P>0.05
Waist -cm	78.53±9.90	90.81±7.62	87.00±10.05	90.83±10.01	85.33±12.74	P>0.05
TG –mmol/l	1.32±0.73	2.21±1.39	1.86±1.09	1.91±1.03	2.44±3.92	P<0.05
TC –mmol/l	5.23±0.80	6.29±2.06	5.29±0.99	5.83±0.98	5.05±1.34	P>0.05

NC, normal controls; PreDM, Prediabetes; DM, diabetes with normal proteinuria; DM-micro, diabetes with microalbuminuria; DM-macro, diabetes with macroproteinuria.

ACR, ablumin-to-creatinine ratio; SBP, systolic blood pressure; DBP, diastolic blood pressure; HR, heart rate; SCr, serum creatinine; Ucr, urine creatinine; BUN, blood urea nitrogen; TG, triglyceride; TC, cholesterol.

### Purity of uEVs by transmission electron microscopy and nanoparticle track analysis

**Figure 2:**
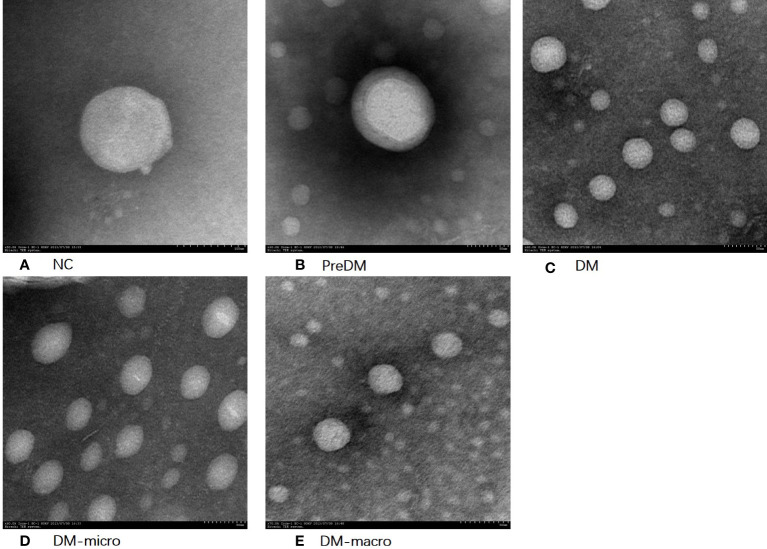
Morphology of uEVs observed by TEM. **(A)**. Morphology of uEVs under TEM in NC. **(B)**. Morphology of uEVs TEM in Prediabetes. **(C)**. Morphology of uEVs under TEM in Diabetes with normal proteinuria. **(D)**. Morphology of uEVs under TEM in Diabetes with microalbuminuria. **(E)**. Morphology of uEVs under TEM in Diabetes with macroproteinuria.

**Figure 3:**
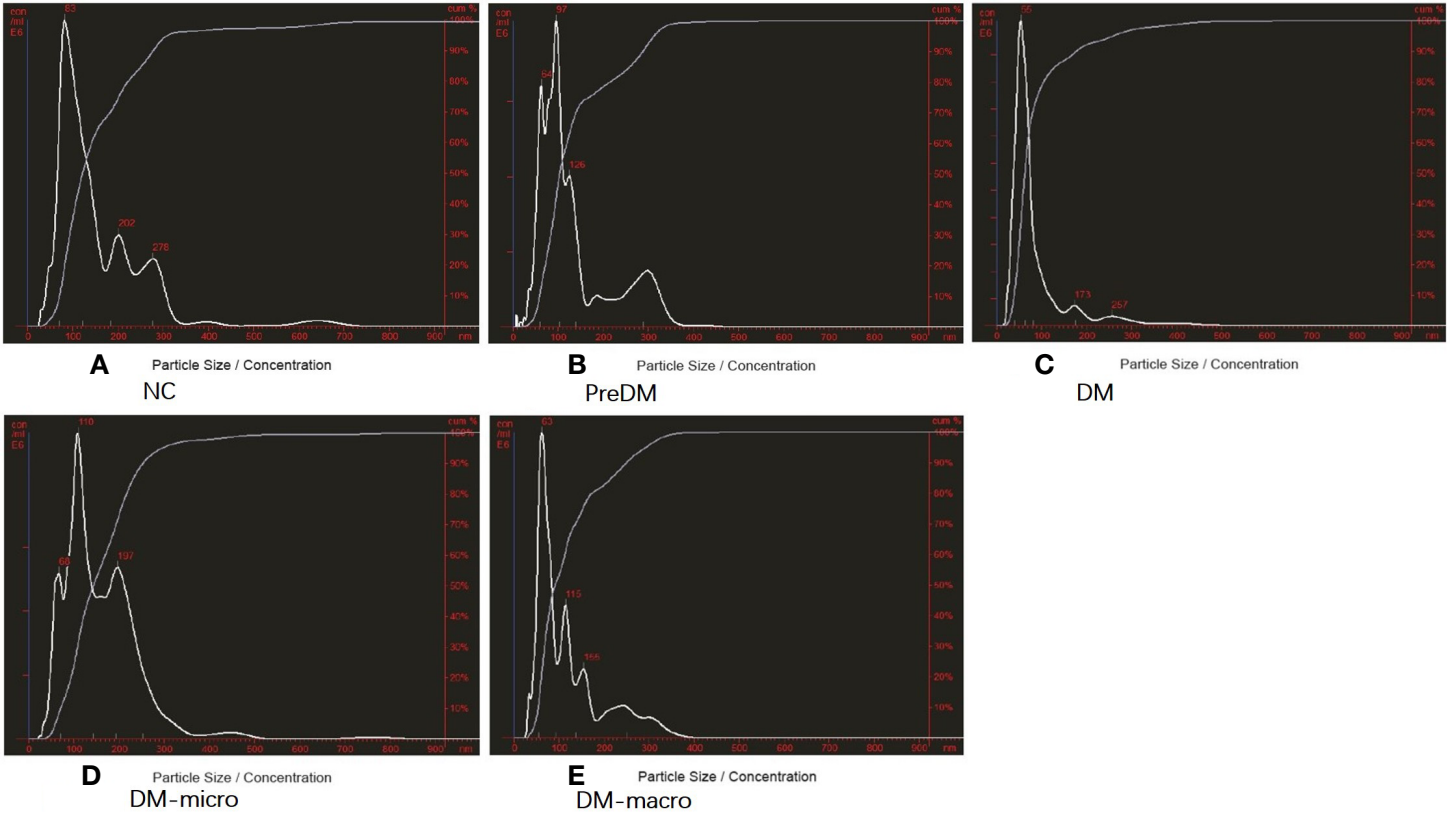
Purity of uEVs by NTA. **(A)**. Purity of uEVs under NTA in NC. **(B)**. Morphology of uEVs under TEM in Prediabetes. **(C)**. Morphology of uEVs under TEM in Diabetes with normal proteinuria. **(D)**. Morphology of uEVs under TEM in Diabetes with microalbuminuria. **(E)**. Morphology of uEVs under TEM in Diabetes with macroproteinuria. NC, normal controls; PreDM, Prediabetes; DM, diabetes with normal proteinuria; DM-micro, diabetes with microalbuminuria; DM-macro, diabetes with macroproteinuria.

The morphology of uEVs from the five groups as observed by TEM is shown in **Figure 2**. Our results indicate that uEVs have a homogeneous distribution, presenting a membrane-encapsulated structure like cup-shaped (**Figure 2B**) or roundish with a diameter of 30–100 nm (**Figures 2A, C, D, E**). A more comprehensive size analysis of uEVs was carried out by NTA on the pool made for each group. The profile showed that the main peak was between 55 and 110 nm, with a predominance of small vesicle in the healthy control (60–80 nm) DM and DM-macro (**Figures 3A, C, E**) and a shift in abundance (100 nm) for PreDM and DM-micro (**Figures 3B, D**). Moreover, some more peaks at a higher diameter were recorded with a characteristic trend for each group (**Figure 3**). All these uEVs under the under the magnification have active Brownian motion and present a membrane-encapsulated structure. In this analysis, all the samples were dialyzed first, and the pellet was then re-suspended in the same buffer eliminating differences in the viscosity of the liquid which can affect the rate of movement.

### Protein concentration of uEVs

**Figure 4:**
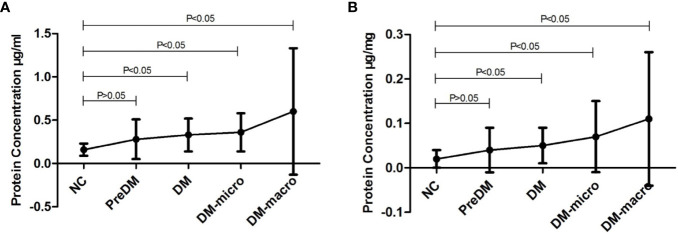
Protein concentration of uEVs in normal controls and diabetes. **(A)**. Protein concentration of uEVs in normal controls and diabetes (Mean±SD). **(B)**. Protein concentration of uEVs in normal controls and diabetes (Mean±SD) adjusted by Ucr.

Data on protein concentration as determined by the Coomassie Bradford assay of uEVs in normal controls and diabetes patients are shown in **Table 2**. The Bradford assay showed that the protein concentration of the five groups differ from each other. The protein concentration of uEVs was 0.16 ± 0.07, 0.28 ± 0.23, 0.33 ± 0.19, 0.36 ± 0.22, and 0.60 ± 0.73 μg/ml, respectively, in normal controls and in prediabetes, diabetes with normal proteinuria, diabetes with microalbuminuria, and diabetes with macroproteinuria groups (**Figure 4A**). There was a higher protein concentration of uEVs in diabetes patients and a higher number of complications than normal controls; however, the protein concentration of uEVs in normal controls and prediabetes did not show a significant difference.

**Table 2:** Protein concentrations of uEVs in normal controls and diabetes (Mean±SD).

	NC	Pre-DM	DM	DM-micro	DM-macro	P value
P40 (μg/ml)	0.16±0.07	0.28±0.23	0.33±0.19	0.36±0.22	0.60±0.73	P<0.05
P40 (μg/mg UCr)	0.02±0.02	0.04±0.05	0.05±0.04	0.07±0.08	0.11±0.15	P<0.05
SN40	0	0	0	0	0	----

Protein concentration = Protein amount (μg) / Original urine volume (ml).

Protein concentration adjusted by Ucr = Protein amount (μg) / urinary creatinine (mg).

P40, Pellet 40,000 after 40,000g centrifugation; SN40, Supernatant 40,000 after 40,000g centrifugation.

After adjusting the protein concentration with urinary creatinine by calculating the EVs-to-creatinine ratio, the protein concentration of uEVs was 0.02 ± 0.02, 0.04 ± 0.05, 0.05 ± 0.04, 0.07 ± 0.08, and 0.11 ± 0.15 μg/mg UCr, respectively, in normal controls and in prediabetes, diabetes with normal proteinuria, diabetes with microalbuminuria, and diabetes with macroproteinuria groups (**Figure 4B**). We found that protein concentration presents the same trend as unadjusted.

According to the NTA report, the size distribution was 162 ± 124, 138 ± 84, 90 ± 7, 163 ± 89, and 123 ± 76 nm in normal controls and in prediabetes, diabetes with normal proteinuria, diabetes with microalbuminuria, and diabetes with macroproteinuria groups. There was significant difference among the five groups (*p* < 0.05). According to the NTA report, the total concentration was 4.0 × E10, 7.16 × E10, 11.6 × E10, 11.8 × E8, and 17.44 × E8 particles/ml (total concentration equals detected concentration multiplied by the dilution factor) in normal controls and in prediabetes, diabetes with normal proteinuria, diabetes with microalbuminuria, and diabetes with macroproteinuria groups. It was significantly increased from prediabetes to diabetes with kidney injury groups (*p* < 0.05).

### Protein pattern and western blot of TSG101 on uEVs

**Figure 5:**
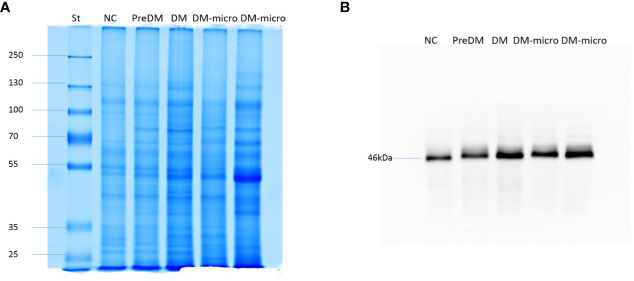
Protein pattern of uEVs and western blot of TSG101. **(A)**. Protein pattern of uEVs on the colloidal gel. **(B)**. Western blot of TSG101 as biogenesis biomarker of uEVs.

In total, 4 μg of exosome protein for each group was loaded on the colloidal gel. The protein pattern of Coomassie brilliant blue G-250 staining revealed that the protein band varied in the different groups (**Figure 5A**). TSG101 is the biogenesis biomarker of eUVs, and the western blot of TSG101 showed that TSG101 was present in all these five groups and exhibit a bit of difference (**Figure 5B**).

## Discussion

Our study was established on the epidemiological survey, and all of the participants were selected from our community-based research (20). The baseline information of normal controls and diabetes patients is listed in **Table 1**, showing that the between-group variations of blood glucose, ACR, SBP, HR, SCr, and TG were significant by one-way ANOVO analysis. Diabetes increased the risk of developing a number of complications, such as neuropathy, nephropathy, retinopathy, and micro- and macro-vascular diseases. Our research focuses on kidney injury; however, there are still some unavoidable confounding factors such as SBP, HR, and TG.

In this study, we introduce a simple method of uEV recovery by hydrostatic filtration dialysis which employs a dry membrane with a molecular weight cutoff of 1,000 kDa to efficiently enrich the uEVs (27). Traditionally, uEVs have been separated by ultracentrifugation. Luca Musante found that the ultracentrifugation method is expensive and has poor efficiency because there are at least 40% of small uEVs that cannot be fully isolated from the supernatant after 200,0000 *g* ultracentrifugation (32). Our hydrostatic filtration dialysis method has the advantage of pre-processing and concentration of samples, the same as the conventional differential centrifugation method and density gradient ultracentrifugation method (27). It is super cost-efficient to enrich the uEVs from urine for clinical application. Since the uEV separation method shows specific advantages and disadvantages, the selected isolation method may play an important role in reflecting the characteristics of isolated EVs and contaminants, and there was not yet a standard operation procedure for their isolation; therefore, we should focus on the purity and yield of uEVs (8).

Decent uEVs were finally obtained in our present study. It has a homogeneous distribution, presents a membrane-encapsulated structure that was cup-shaped or roundish under TEM, shows active Brownian motion, and presents the main peak between 55 and 110 nm under NTA. All these uEVs under the magnification field show active Brownian motion and present a membrane-encapsulated structure by NTA analysis. The roundish morphology observed under TEM may be caused by our phosphotungstic acid negative staining method instead of the more common negative staining based on uranyl acetate. The diameter of uEVs under TEM and NTA has some difference, which may be due to the fact that NTA observes the hydrodynamic particle diameter of the uEVs through a liquid medium; another is that larger particles contribute more strongly to dynamic light scattering than the smaller ones (33). According to the NTA report, the size distribution was significantly different among the five groups. This difference may be caused by aggregation of the microspheres, optical alignment, polydisperse in preparation procedure, *etc.* However, detailed calibration—different dilution factors of 1:1,600 in diabetes with macroproteinuria group and 1:200 in the rest of the groups—was carried out during the identification procedure in order to avoid such confounders. Another question is that there was no standard for uEV measurement. Furthermore, the size of our vesicles ranged from 30 to 500 nm according to the characteristic of EVs, and the total concentration was significantly increased from prediabetes to diabetes with kidney injury groups.

There were three centrifugation methods popular in EV extraction, such as low-force centrifugation at less than 10,000 *g*, ultracentrifugation that varies from 100,000 to 200,000 *g*, and differential centrifugation steps including both of those previously mentioned. With the progress in technology of EV preparation, P21 (pellet after 21,130 *g* centrifugation) was proved to have the rigorous characteristics of uEVs by Luca Musante and his team (31). After hydrostatic filtration dialysis and a relatively low-force centrifugation of 40,000 *g* in our study, P40 presents the biogenic characteristics of uEVs under TEM and NTA. There are couples of biomarkers of EVs. TSG101 was the biogenesis biomarker of uEVs (34) and expressed throughout the urogenital tract, having the nature for uEV identification. The western blot of TSG101 confirmed our urinary uEVs. Therefore, P40 is suitable for clinical application.

Our protein assay result suggests that there is a significant difference between normal controls and diabetes. After adjustment by UCr, the adjusted uEV concentrations still vary a lot between the disease cases and the normal controls. Therefore, uEVs may vary in disease cases to healthy individuals. Unfortunately, the lack of a housekeeping protein does not allow the precise normalization of the vesicle quantity as well as any other protein target carried by uEVs. Moreover, other than diabetes and its complication, several variables such as age, gender, diet, nutritional status, physical activity, collection time, volume status, environmental factors, *etc.*, might have impact on uEV secretion, thus resulting in a discrepancy between the two groups of participants (24, 35). The UCr concentration has the natural ability of a normalizer for uEV concentration because of its correlation with particle number and its excretion being not affected by water intake (36, 37). Concerning these confounding factors, UCr was used to adjust the protein concentration of uEVs as recommended (24) in order to have better quantification. However, the adjusted uEV protein concentration remains in the same trend. Therefore, the uEV-to-UCr ratio may be considered as a housekeeper indicating the stage of DN. uEVs may be non-invasive biomarkers in predicting and monitoring the progression of renal physiological and pathological conditions (38, 39).

More and more evidence have established a valuable role of uEVs in renal physiology and pathology (38, 40–42). A study focused on the interaction of the glomerular endothelial-derived EVs and podocytes established the central role of EV-mediated communication in playing a negative effect on podocyte function (40). Moreover, podocyte-derived EVs might establish a crosstalk between glomeruli and tubules and impair tubular epithelial cell by initializing an apoptosis program (41). That EVs could mediate long-distance cell-to-cell communication has already been proven. This might promote tubulointerstitial fibrosis and aggravate pathological progression, thus amplifying the damage of the kidney (43). However, whether the protein content in uEVs faithfully reflects the characteristics of renal cells and tissue or not is still a subject in debate. An animal study established a significant correlation between uEVs and renal protein abundance by proteomic analysis (34). A recent study revealed that the abundance of phosphorylated sodium chloride cotransporter in uEVs was significantly higher (1.89 folds) than that of the controls. Therefore, the protein biomarker of uEVs may be considered as an indicator of adrenal venous sampling (44). Our results also established the reliability of using uEV protein changes to monitor physiological and pathological responses in diabetic nephropathy.

In short, a simple method called hydrostatic filtration dialysis was used to enrich uEVs in our study, and it revealed that the abundance and cargo of uEVs vary in diabetes with or without kidney injury, which may be involved in the physiological and pathological changes of diabetes. However, the different techniques used to isolate EV subtypes and EVs from complicated components may result in preparations with different levels of abundance and purity of EVs (45). Our study revealed that the uEV-to-UCr ratio may have advantage over urine protein–creatinine ratio or albumin–creatinine ratio, which may indicate different stages of DN. Furthermore, it has cost-effectiveness in terms of patient management. However, further research still needs to be developed to prove this.

## Data availability statement

The original contributions presented in the study are included in the article/supplementary material. Further inquiries can be directed to the corresponding author.

## Ethics statement

The studies involving human participants were reviewed and approved by the Ethics Committee of the Third Affiliated Hospital of Southern Medical University. The patients/participants provided their written informed consent to participate in this study.

## Author contributions

DG performed the experiment and interpreted the data. DG and YD drafted the manuscript. XJ and BS collected the samples and epidemiological data. LM initiated the method of uEV enrichment. HZ and HH designed the study and reviewed the manuscript. All authors contributed to the manuscript and approved the submitted version.
